# Genomic Analysis of a Novel *Torradovirus* “Rehmannia Torradovirus Virus”: Two Distinct Variants Infecting *Rehmannia glutinosa*

**DOI:** 10.3390/microorganisms12081643

**Published:** 2024-08-11

**Authors:** Yanhong Qin, Shuhao Lu, Yi Wen, Shaojian Li, Suxia Gao, Desheng Zhang, Xuemeng Li, Jin Yang, Li Gu, Mingjie Li, Fei Wang, Chuantao Lu

**Affiliations:** 1 Institute of Plant Protection, Henan Academy of Agricultural Sciences, No. 116, Huanyuan Road, Jinshui District, Zhengzhou 450002, China; qinyanhong6040@163.com (Y.Q.); 13629840525@163.com (S.L.); wy412@163.com (Y.W.); lishaojianli@126.com (S.L.); gaosx78@126.com (S.G.); zhangdesheng404@163.com (D.Z.); limm022@163.com (X.L.); yangjj0621@163.com (J.Y.); wangfei@hnagri.org.cn (F.W.); 2 College of Crop Sciences, Fujian Agriculture and Forestry University, Fuzhou 350002, China; guli5101@163.com (L.G.); xinyuzszj@163.com (M.L.)

**Keywords:** *Rehmannia glutinosa*, Rehmannia torradovirus virus, *Torradoviruses*, high-throughput sequencing, sequence comparison, phylogenetic relationship

## Abstract

*Rehmannia glutinosa*, a crucial medicinal plant native to China, is extensively cultivated across East Asia. We used high-throughput sequencing to identify viruses infecting *R. glutinosa* with mosaic, leaf yellowing, and necrotic symptoms. A novel *Torradovirus,* which we tentatively named “Rehmannia torradovirus virus” (ReTV), was identified. The complete sequences were obtained through reverse-transcription polymerase chain reaction (RT-PCR), 5′ and 3′ rapid amplification of cDNA ends, and Sanger sequencing. The amino acid sequence alignment between the ReTV-52 isolate and known *Torradovirus* species in the Pro-Pol and coat protein regions were 51.3–73.3% and 37.1–68.1%, respectively. Meanwhile, the amino acid sequence alignment between the ReTV-8 isolate and known *Torradovirus* species in the Pro-Pol and coat protein regions were 52.7–72.8% and 36.8–67.5%, respectively. The sequence analysis classified ten ReTV strains into two variants. The ReTV-52 genome has two RNA segments of 6939 and 4569 nucleotides, while that of ReTV-8 consists of two RNA segments containing 6889 and 4662 nucleotides. Sequence comparisons and phylogenetic analysis showed ReTV strains clustered within the *Torradovirus,* exhibiting the closet relation to the squash chlorotic leaf spot virus. The RT-PCR results showed a 100% ReTV detection rate in all 60 *R. glutinosa* samples. Therefore, ReTV should be classified as a novel *Torradovirus* species. ReTV is potentially dangerous to *R. glutinosa,* and necessitating monitoring this virus in the field.

## 1. Introduction

*Rehmannia glutinosa* (family Scrophulariaceae) is an economically significant herbaceous medicinal plant. It is indigenous to China and widely cultivated in China, Korea, Japan, and northern Vietnam [1]. The fresh or dried root tubers of *R. glutinosa* are used as medicine, and the tuberous roots of *R. glutinosa* are rich in medicinal compounds, such as phenylethanoid glycosides, iridoids, ionones, polysaccharides, flavonoids, sugars, and other components. These active compounds exhibit diverse pharmacological effects on the immune, cardiovascular, blood, endocrine, and nervous systems [2]. *Rehmannia glutinosa* is the second most commonly used among 203 Chinese patent medicine prescriptions, and is one of the top 10 species used in Chinese herbal medicines and products exported from China. In China, *R. glutinosa* is one of the famous “Four Huai Medicines” and is grown in a significant cultivation area exceeding 1000 hectares in northern China [3]. The primary production regions include the Henan, Shanxi, Shandong, and Hebei Provinces. Jiaozuo City in Henan Province stands out as a major production region. Catapol, rehmannioside A, and rehmannioside D contents of *R. glutinosa* from this area are the highest among plants from all regions [4].

*Rehmannia glutinosa* is conventionally propagated through tuberous roots rather than seeds. This method of vegetative propagation often has a high incidence of viral diseases in the field. This leads to significant losses in yield and quality in the primary producing regions of China [5]. Globally, twelve viruses have been found to infect *R. glutinosa*, including Rehmannia mosaic virus (ReMV), broad bean wild virus 2 (BBWV2) [6], tobacco mosaic virus (TMV), cucumber mosaic virus (CMV), tomato mosaic virus (ToMV) [7], Plantago asiatica mosaic virus [8], ribgrass mosaic virus (RMV) [1], youcai mosaic virus (YoMV) [9], Rehmannia virus 1 [10], Cucurbit chlorotic yellows virus (CCYV) [11], tobacco mild green mosaic virus (TMGMV) [12], Rehmannia allexivirus virus (ReAV) [13], and columnea latent viroid (CLVd) [14]. In China, ten viruses—ReMV, CMV, BBWV2, TMV, ToMV, YoMV, CCYV, TMGMV, CLVd and ReAV—have been identified as infecting *R. glutinosa* [4,11,12,13,14]. In field conditions, *R. glutinosa* was infected with one or more of these viruses, exhibiting symptoms such as mosaic patterns, chlorosis, necrotic spots, vein necrosis, yellowing, and stunted growth.

*Torradoviruses* are classified with the family *Secoviridae* under the order *Picornavirales* [15]. They were first identified in 2007 as two novel virus species: tomato torrado virus (ToTV) and tomato marchitez virus (ToMarV) [16,17]. Natural *Torradovirus* infections were initially described in tomatoes (*Solanum lycopersicum* L.). Subsequently, other *Torradoviruses* were recognized in non-tomato plants; with the development of new tools, several other host plants have been reported, including carrot [18], lettuce [19], motherwort [20], squash [21], cassava [22], and burdock [23]. According to the latest International Committee on Taxonomy of Viruses (ICTV) report, Master Species List 39 (MSL39), this genus encompasses nine member species: ToTV, ToMarV, carrot torradovirus 1 (CaTV1), lettuce necrotic leaf curl virus (LNLCV), motherwort yellow mottle virus (MYMoV), squash chlorotic leaf spot virus (SCLSV), codonopsis torradovirus A (CoTVA), cassava torrado-like virus (CsTLV), and fleabane yellow mosaic virus (FbYMV) [24]. In addition to the nine viruses previously mentioned, tomato chocolate virus (ToChV), tomato chocolate spot virus (ToChSV) [25], and tomato necrotic dwarf virus (ToNDV) have been suggested as a tentative species of the genus *Torradovirus*. *Torradoviruses* are composed of small spherical virions measuring approximately 30 nm in diameter. Their genome includes two linear positive-sense single-stranded RNA molecules of approximately 7 and 5 kb, respectively. RNA1 carries a single large open reading frame (ORF) that encodes a putative polyprotein comprising conserved helicase, protease, and RNA-dependent RNA polymerase (RdRp). RNA2 has two predicted ORFs encoding two polyproteins: RNA2-ORF1 encodes a putative polyprotein of unknown function, while ORF2 encodes a polyprotein that contains a putative movement protein (MP) at its N, followed by three coat proteins (CPs). In species classification, two prevailing criteria for demarcating members of the *Secoviridae* family include amino acid (aa) sequence identities of <80% in the Pro-Pol region (the “CG” motif of the 3C-like protease and the “GDD” motif of the RNA-dependent RNA polymerase) of RNA1-ORF1 and <75% in the CP regions of RNA2-ORF2. Previous comparisons within this genus have distinguished two groups: tomato-infecting (TI) and non-tomato-infecting (NTI) members, based on the aa sequence identities of encoding putative protein and the length of 3′ UTRs [26].

In this study, a putative novel virus that severely affects *R. glutinosa* in the field, influencing its growth. The virus was named “rehmannia torradovirus virus (ReTV)”. The whole genome sequences of ReTV were determined. The genome organization, phylogenetic relationships, and molecular variations of the virus were analyzed. It is proposed ReTV to be a new member of the genus *Torradovirus*. 

## 2. Materials and Methods

### 2.1. Plant Material

Sixty *R. glutinosa* samples exhibiting virus-like symptoms such as mosaic, yellowing, mottling, and necrosis were collected between June and July 2020. Each sample consisted of two or three leaves from an individual plant. These samples—Wenxian (23/60), Wuzhi (20/60), and Yuzhou (17/60)—were obtained from three locations in Henan Province, China. To investigate the presence of viral agents associated with these virus-like symptoms, high-throughput sequencing (HTS) was performed on all 60-leaf samples of *R. glutinosa*. Before the HTS, all samples were ground in liquid nitrogen and individually stored at −80 °C.

### 2.2. High-Throughput Sequencing Analysis

To identify potential viruses in the samples, small portions of each collected leaf sample were combined into a mixed sample and sent to Berry Genomics Corporation (Beijing, China) for HTS analysis. Total RNA was extracted from all 60 leaf samples using the RNAprep Pure Plant Plus kit (Tiangen, Beijing, China). RNA quantity and quantity were assessed using a Nanodrop 2000 analyzer and agarose gel electrophoresis, respectively. The transcriptome library was constructed using the NEBNext Ultra RNA Library Prep Kit from Illumina (San Diego, CA, USA). Sequencing was performed using the Illumina Nova Seq6000 sequencing system (Berry Genomics Corporation, Beijing, China). The processing and analysis of the sequencing data were completed by Wuhan Biowefind Co., Ltd. (Wuhan, China), who mainly performed the processing and splicing of the sequencing data. The raw reads were trimmed of adapter sequences and filtered for low-quality reads using FASTP version 1.5.6c [27]. The trimmed reads were de novo assembled into larger contigs using IDBA-UD version 1.1.1 [28] with k-mer values of 80, 90, and 110. The resulting contigs were aligned to the protein database using a BLASTx search on NCBI (https://www.ncbi.nlm.nih.gov/ (accessed on 5 June 2024)).

### 2.3. The Full Genome Assembly of ReTV and Sequence Analysis

At an early stage, the results of HTS showed that contigs contained two ReTV variants. From 60 *R. glutinosa* samples, ten strains were randomly selected to obtain the full sequences of ReTV variants, for which specific primers were designed based on the contigs of HTS results (Supplementary Table S1). The primers were synthesized by Sangon Biotech Co., Ltd. (Shanghai, China). Total RNA from positive samples was extracted using a Spin Column Plant Total RNA Purification Kit (Sangon Biotech, Shanghai, China). Single-stranded cDNA was synthesized using the PrimeScript^TM^ II 1st Strand cDNA Synthesis kit (Takara, Dalian, China), with RNA serving as the template, and the cDNA was stored at −20 °C.

The overlapping fragments covering the genomic sequence of ReTV were amplified by RT-PCR. The PCR reaction system consisted of the following: 2× *taq* Master Mix, 10 μL; forward primer (10 μM), 0.5 μL; reverse primers (10 μM), 0.5 μL; cDNA, 1 μL; and ddH_2_O added to create a final volume of 20 µL. PCR conditions included an initial denaturation at 95 °C for 5 min, 35 cycles of denaturation at 95 °C for 30 s, annealing at 55 °C for 30 s, extension at 72 °C for 1–2 min, and a final extension step at 72 °C for 10 min. The mixed samples were then stored at −20 °C. The sequences of the 5′ and 3′ ends were obtained by rapid amplification of cDNA ends (RACE) using a SMARTer RACE 5′ and 3′ Kit (Sangon Biotech), respectively. All amplicons were recovered, purified, cloned into the pMD19-T vector (Takara), transformed into competent *Escherichia coli* TG1 cells, and subsequently sequenced.

All overlapping sequence fragments were analyzed and assembled using DNAMAN 7.0. ORFs for the entire sequence were predicted using the ORF Finder on the NCBI for Biotechnology Information website (https://www.ncbi.nlm.nih.gov/orffinder/ (accessed on 10 June 2024)). Subsequently, the full sequences were submitted to GenBank (OR453958-OR453977). The motif “Helicase, 3C-like protease, and RdRp” was predicted using ScanProsite (https://prosite.expasy.org/scanprosite/ (accessed on 12 March 2024)). Sequence alignment and homology analyses for the complete ReTV genome and its encoded genes were performed by each functional protein using DNAMAN 7.0.

### 2.4. Phylogenetic Analysis

To identify species and calculate similarity percentages, the amino acid sequences of the Pro-Pol and CP genes of ten ReTV strains from this study were selected, together with those of members of the four genera (*Torradovirus*, *Sadwavirus*, *Stralarivirus*, and *Cheravirus* ) belonging to the family *Secoviridae* from NCBI. *Torradovirus* includes MYMoV, CoTVA, LNLCV, CaTV1, ToMarV, ToCSV, ToTV, SCLSV, and CsTLV. *Cheravirus* includes cherry rasp leaf virus (CRLV), apple latent spherical virus (ALSV), and currant latent virus (CuLV). *Sadwavirus* includes satsuma dwarf virus (SDV). Finally, *Stralarivirus* includes *Lychnis* mottle virus (LycMoV) and strawberry latent ringspot virus (SLRSV). Additional specific information on these species is provided in Supplementary Table S2. Thirty-nine sequences were aligned using MEGA11.0 software [29]; the phylogenetic tree was estimated using the maximum likelihood method and a Poisson model with a bootstrap value of 1000.

### 2.5. Recombination Analysis of Ten ReTV Isolates and Other Torradoviruses

The complete genome sequences of the ReTV strains (OR453958-OR453977) and other Torradoviruses (including RNA1 and RNA2) that were same as those listed in Table 1 used for sequence comparisons were aligned using Clustal X1 [30]. Alignment results were analyzed using Recombination Detection Program (RDP) v.4.101 software [31]. The following analytical methods were employed: RDP, GENECONV, BootScan, MaxChi, Chimacra, SiScan, and 3Seq. Default parameters were applied for each program during recombination detection. Recombination was identified in the RDP analysis when three or more methods detected it, with a *p*-value below 10^−5^ indicating a significant recombination event for each method.

### 2.6. RT-PCR Detection of ReTV in R. glutinosa Samples

Total RNA was extracted, followed by reverse transcription, and PCR amplification was performed. The PCR products were separated using agarose gel electrophoresis, and their sizes were confirmed with an ultraviolet lamp and agarose gel imaging system. The PCR products were purified using the column method (E.Z.N.A. Gel Extraction kit, Omega Bio-tek, Norcross, GA, USA) for Sanger sequencing. Molecular variations were assessed after aligning the nucleotide sequences using DNAMAN software7.0 [32].

## 3. Results

### 3.1. High-Throughput Sequencing Analysis Data

HTS of the RNA-seq library yielded 28,522,540 raw reads, totaling over 8 GB. After trimming, 27,664,949 high-quality clean reads were obtained and used for contig assembly. These assembled contigs underwent a local BLAST for a BLASTx search in GenBank. All 12 contigs were similar to *Torradovirus*-like viruses. Among these, three large contigs of 6716, 4782, and 5145 nt shared 51.05, 57.40, and 54.31% aa sequence identity with the highest match to RNA1 of SCLSV (GenBank accession number: UZN89714) of the genus *Torradovirus*, respectively. Two contigs of 3828 and 3753 nt shared 60.36% and 60.75% aa sequence identity, respectively, with the highest match to RNA2 of SCLSV (GenBank accession number: UZN89715) of the genus *Torradovirus*.

### 3.2. Genome Organization of ReTV

To acquire complete sequences of ReTV, twenty sequences were obtained through RT-PCR, 5′ and 3′ RACE of RNA1 and RNA2, including four complete and sixteen nearly full sequences. Upon comparing these sequences, two distinct variants—ReTV-52 and ReTV-8, named “rehmannia torradovirus virus (ReTV)”—were identified.

**Figure 1:**
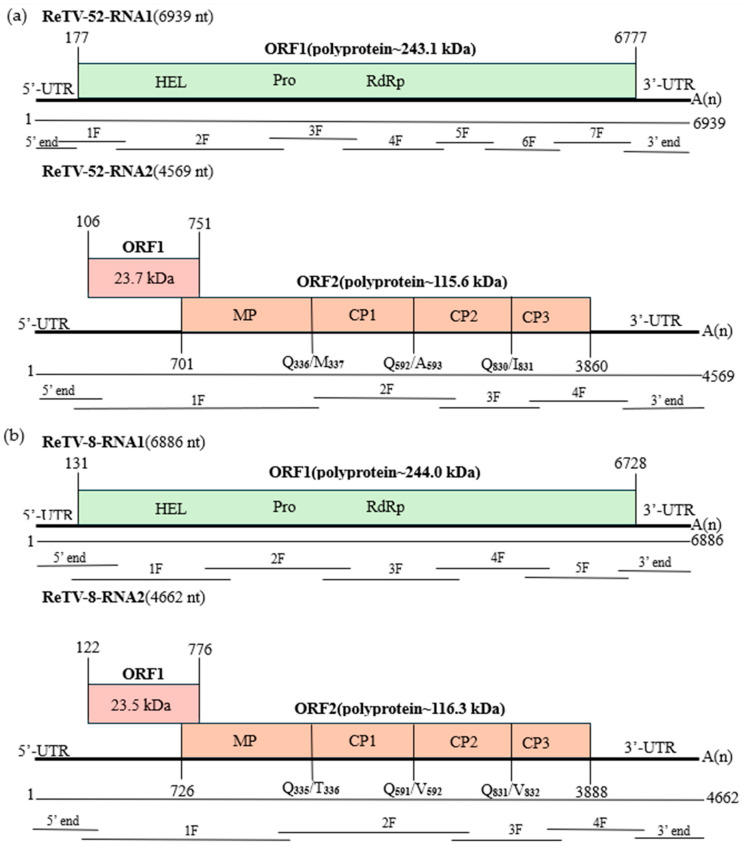
Genome organization of the rehmannia torradovirus virus (ReTV) showing relative positions of ORFs and their expression products. (**a**) rehmannia torradovirus virus-52, (**b**) rehmannia torradovirus virus-8. The RNA1 indicated the positions of sequences encoding conserved protein domains (HEL, Pro, and RDRP), while the RNA2 indicate the putative cleavage sites for the MP and CPs. The molecular weight predicted for each protein is reported above the boxes. RNA1 and RNA2 have indicated the start and stop positions of each virus segment in the viral genome organization.

**Figure 2:**
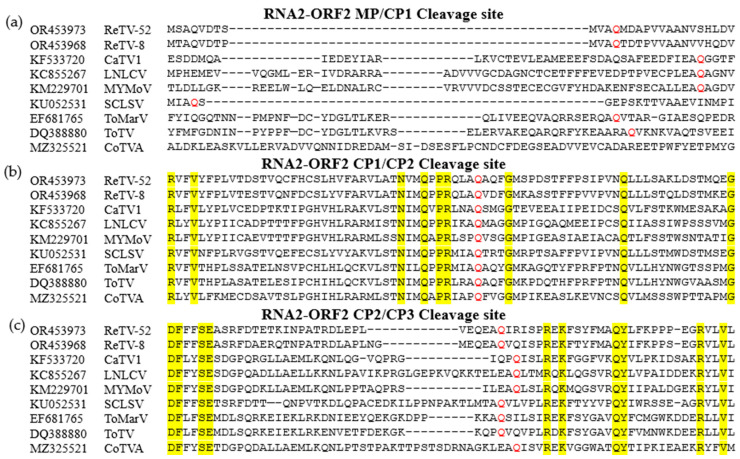
Cleavage sites of RNA2-ORF2. (**a**) RNA2-ORF2 MP/CP1 cleavage site, (**b**) RNA2-ORF2 CP1/CP2 cleavage site, and (**c**) RNA2-ORF2 CP2/CP3 cleavage site. Putative conserved glutamine (Q) at the −1 position of the cleavage site is highlighted in red. Identical bases are highlighted in yellow. ORF, open reading frame.

The full-length RNA1 of ReTV-52 measured 6939 nt (GenBank accession number: OR453962), featuring a poly(A) tail at the 3′ terminal. It included a single ORF from 177–6777 nt, encoding a polyprotein of 2200 aa with a predicted molecular mass of 243.1 kDa (Figure 1a). Three conserved motifs were identified using ScanProsite: a helicase (PS51218) at positions 368–538 aa, a 3C-like protease (PS51874) at positions 892–1113 aa, and RdRp (PS50507) at positions 1403–1538 aa. The full-length RNA2 of ReTV-52 is 4569 nt (GenBank accession number: OR453973), featuring a poly(A) tail at the 3′ terminal. It contained two ORFs with overlapping regions (nt 701–751). The first ORF from 106–751 nt encodes a protein of 215 aa with a predicted molecular mass of 23.7 kDa. The second ORF from 701–3861 nt encodes a polyprotein of 1053 aa with a predicted molecular mass of 115.6 kDa. The polyprotein is thought to be cleaved into an MP and three CPs. The cleavage sites—Q_336_/M_337_, Q_592_/A_593_, and Q_830_/I_831_—were identified through the alignment of the aa sequences with those of other *Torradoviruses* (Figure 2).

The full-length RNA1 of ReTV-8 is 6889 nt (GenBank accession number: OR453963), with a poly (A) tail at the 3′ terminal. It includes a single ORF from 131–6728 nt, encoding a polyprotein of 2199 aa with a predicted molecular mass of 244.0 kDa (Figure 1b). Three conserved motifs were identified using ScanProsite website: a helicase (PS51218) at positions 368–538 aa, a 3C-like protease (PS51874) at positions 888–1111 aa, and RdRp (PS50507) at positions 1401–1536 aa. The full-length RNA2 of ReTV-8 is 4662 nt (GenBank accession number: OR453968), with a poly(A) tail at the 3′ terminal. It contains two ORFs with overlapping regions (nt 726–776). The first ORF from 122–776 nt encodes a protein of 218 aa with a predicted molecular mass of 23.5 kDa. The second ORF from 726–3888 nt encodes a 1054-aa polyprotein with a predicted molecular mass of 116.3 kDa. The polyprotein is thought to be cleaved into an MP and three CPs. The cleavage sites—Q_335_/T_336_, Q_591_/V_592_, and Q_831_/V_832_—were identified through the alignment of the aa sequences with those of other *Torradoviruses* (Figure 2). 

### 3.3. Sequence Comparisons of ReTV with Other Torradoviruses

To determine the relationship of ReTV-52 and ReTV-8 within the genus *Torradovirus*, their aa sequences were compared with those of ten *Torradovirus* members (Table 1). These members include MYMoV, CaTV1, Codonopsis torradovirus A (CoTVA), SCLSV, LNLCV, ToTV, ToMarV, burdock mosaic virus (BdMV), FbYMV, and ToChSV. The comparative analysis across different regions indicated that the homology for RNA1-ORF1 ranged from 31.7–54.6% and 32.2–54.9% for ReTV–52 and ReTV–8, respectively. For the Pro-Pol regions, aa homology ranged from 51.3–73.3% and 52.7–72.8% for ReTV–52 and ReTV–8, respectively. For RNA2-ORF1, aa homology ranged from 18.2–47.0% and 22.1–47.5% for ReTV–52 and ReTV–8, respectively. The polyprotein encoded by RNA2-ORF2 exhibited aa homology ranging from 31.4–62.2% and 31.5–62.2% for ReTV–52 and ReTV–8, respectively. The MP displayed aa homology range of 22.7–52.3% and 23.3–54.3% for ReTV–52 and ReTV–8, respectively. The three CPs had aa homology values in the ranges of 37.1–68.1% and 36.8–67.5% for ReTV–52 and ReTV–8, respectively. In summary, ReTV–52 and ReTV–8 exhibited the highest aa sequence homology with SCLSV strain Su12-10 (GenBank accession number: KU052530/KU052531). According to the species classification criteria, two prevailing demarcation criteria for members of *Secoviridae* include aa sequence identities <80% in the Pro-Pol region of RNA1-ORF1 and <75% in the CP regions. The highest aa sequence identities observed for ReTV–52 and ReTV–8 in the Pro-Pol and CPs regions were 73.3/72.8% and 68.1/67.5% to SCLSV strain Su12-10, respectively. Thus, the maximum levels observed were below the species threshold. Consequently, based on these results, it is proposed that this novel virus be classified as a tentative member of a new species within the genus *Torradovirus* in accordance with the demarcation criterion. This marks the first identification of a virus within the genus *Torradovirus* infecting R. *glutinosa*.

### 3.4. Molecular Variation of ReTV Genome Sequences

To investigate the molecular variations in ReTV, we analyzed the genome sequences of ten isolates from *R. glutinosa*. Table 2 shows the nucleotide sequence comparison of RNA1 and RNA2 with ReTV isolates, indicating that the ten ReTV isolates likely belonged to two variants. The first variant including ReTV-40, ReTV-41, ReTV-44, ReTV-52, and ReTV-53 displayed >91.7% and >93.8% nucleotide homologies with RNA1 and RNA2, respectively. The second variant including ReTV-7, ReTV-8, ReTV-39, ReTV-51, and ReTV-57, exhibited >99.2% nucleotide homology with RNA1 and >99.3% nucleotide homology with RNA2. We also compared the aa sequences of the Pro-Pol regions and CPs with those of the ten ReTV isolates (Table 3). The Pro-Pol comparison results showed that ReTV-7, ReTV-8, ReTV-39, ReTV-51, and ReTV-57 exhibited aa homology of 98.1–99.6%. However, they displayed aa homology of 83.2–84.1% with ReTV-40, ReTV-41, ReTV-44, ReTV-52, and ReTV-53. The CP comparison results showed that ReTV-7, ReTV-8, ReTV-39, ReTV-51, and ReTV-57 displayed aa homology of 99.2–99.4%. However, they displayed aa homology of 82.6–83.7% with ReTV-40, ReTV-41, ReTV-44, ReTV-52, and ReTV-53. Based on the species classification criteria, we considered the ten ReTV isolates to be the same virus but separated into two variants due to the highly significant differences.

### 3.5. Recombination Analysis of the Ten ReTV Strains and Other Torradoviruses

**Figure 3:**
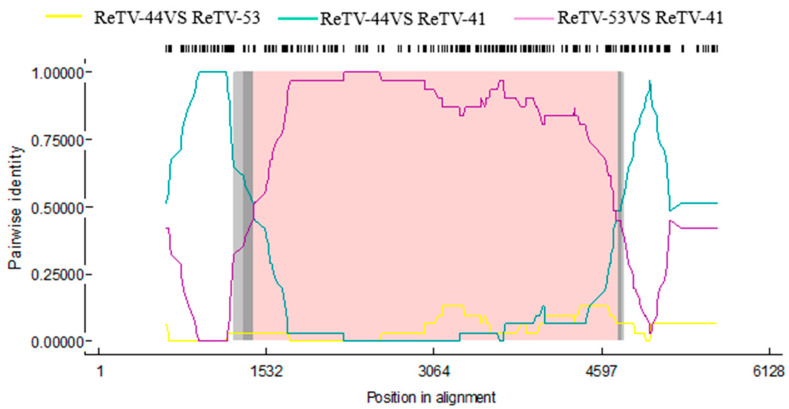
Recombination analysis of ReTV-41 isolates using the recombination detection program RDP4.1 Dark gray regions represent a 95% breakpoint confidence interval, light gray region indicates a 99% breakpoint confidence interval, while the pink region highlights a tract of sequence with a recombination origin. ReTV, rehmannia torradovirus virus.

Recombination drives viral evolution and novel virus production. To examine the evolution of the ten ReTV strains and other *Torradoviruses* (MYMoV, ToTV, CaTV1, SCLSV, LNLCV, CsTLV, CoTVA, FbYMV, and BdMV ), it was employed RDP4.1 software to perform recombination analysis of RNA1 and RNA2. The results showed that there was no recombination in the ReTV-RNA1s. However, ReTV-41 underwent recombination with ReTV-RNA2, with ReTV-44 strains as the major parent and ReTV-53 as the minor parent. Figure 3 shows minor recombination potential at positions 1406-4754 nt. The six analysis methods—RDP, GENECONV, BootScan, MaxChi, SiScan, and 3Seq—confirmed this recombination (*p* < 10^−5^), with *p*-values of 3.644 × 10^−12^, 2.676 × 10^−17^, 3.646 × 10^−12^, 3.903 × 10^−15^, 3.450 × 10^−29^, and 1.431 × 10^−29^, respectively. These results show that the obtained ReTV-41 is a recombinant ReTV strain from ReTV-44 and ReTV-53.

### 3.6. Phylogenetic Analysis of ReTV Strains

**Figure 4:**
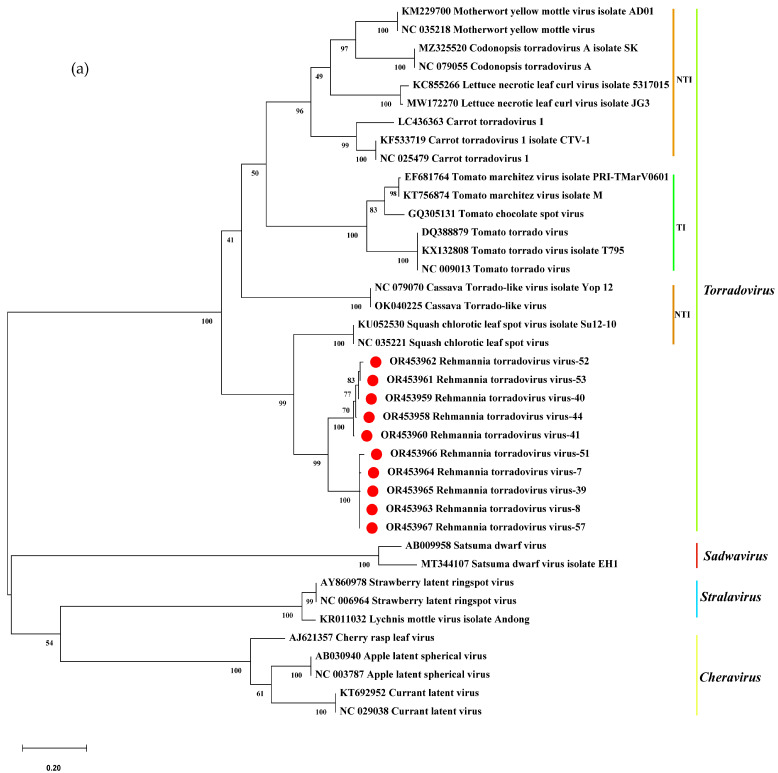

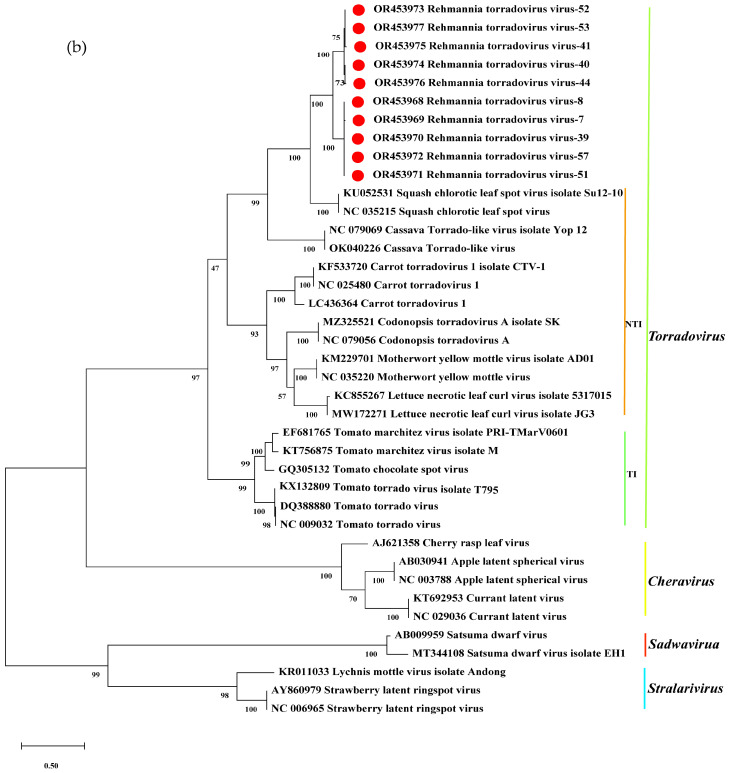
Phylogenetic analysis of ReTV and representative members of the family *Torradovirus* based on the amino acid sequence of (**a**) the Pro-Pol regions and (**b**) the CP-containing regions. The phylogenetic trees were constructed using the maximum likelihood method with 1000 bootstrap replications. Red dots: Sequences obtained in this study, TI, tomato-infecting, NTI, non-tomato-infecting. Motherwort yellow mottle virus (MYMoV), Codonopsis torradovirus A (CoTVA), lettuce necrotic leaf curl virus (LNLCV), carrot torradovirus 1 (CaTV1), tomato marchitez virus (ToMarV), tomato chocolate spot virus (ToCSV), tomato torrado virus (ToTV), cassava torrado-like virus (CsTLV), squash chlorotic leaf spot virus (SCLSV), cherry rasp leaf virus (CRLV), apple latent spherical virus (ALSV), currant latent virus (CuLV), satsuma dwarf virus (SDV), lychnis mottle virus (LycMoV), and strawberry latent ringspot virus (SLRSV).

To further assess the taxonomic position of ReTV, the aa sequences of Pro-Pol and CPs of ReTV were aligned with other torradoviruses using the ClustalW algorithm in MEGA 11.0. A bootstrap value of 1000 was utilized to construct a phylogenetic tree using the maximum likelihood method. The results showed that the ten ReTV strains were closely related to the SCLSV strain Su12-10 (GenBank accession number: KU052530) and were categorized into two variants based on the Pro-Pol regions (Figure 4a), and the ten ReTV strains were closely related to the SCLSV strain Su12-10 (GenBank accession number: KU052531) and were categorized into two variants based on the CP regions (Figure 4b). All *torradoviruses* were clustered into two groups (NTI and TI). In conclusion, phylogenetic tree analysis based on Pro-Pol regions and CPs demonstrated the highest homology with SCLSV strain Su12-10. Consequently, the ReTV strains in this study were classified within the NTI group and clustered together with SCLSV and relatives.

### 3.7. RT-PCR Detection of ReTV in R. glutinosa Samples

To investigate the occurrence of ReTV in *R. glutinosa,* leaf samples from sixty plants were collected. Eight primer pairs were employed to detect ReTV1 and ReTV2 in these samples using RT-PCR. Fourteen samples tested positive for ReTV-variant1-RNA1-1F/1R, yielding a detection rate of 23.3%. Nine PCR products were randomly selected for sequencing, revealing a molecular variation between 99.1 and 100%. Fifteen samples tested positive for ReTV-variant1-RNA1-2F/2R, showing a detection rate of 25%. Ten PCR products were randomly selected for sequencing, demonstrating molecular variation from 90.6–99.8%. Sixteen samples tested positive for ReTV-variant1-RNA2-1F/1R, achieving a detection rate of 26.7%. Ten PCR products were selected randomly for sequencing, revealing molecular variation between 93.3 and 100%. Twenty-one samples tested positive for ReTV-variant1-RNA2-2F/2R, with a detection rate of 35%. Ten PCR products were randomly selected for sequencing, showing molecular variations ranging from 92.8–99.8%. All sixty samples tested positive for ReTV-variant2-RNA1-1F/1R, yielding a detection rate of 100%. Seven PCR products were randomly selected for sequencing, revealing molecular variation of 99.8–100%. Similarly, all sixty samples were positive for ReTV-variant2-RNA1-2F/2R, with a detection rate of 100%. Eight PCR products were randomly selected for sequencing, exhibiting molecular variation from 98.0–100%. Furthermore, all sixty samples tested positive for ReTV-variant2-RNA2-1F/1R, with a detection rate of 100%. Eight PCR products were randomly selected for sequencing, showing molecular variation between 99.4% and 100%. Fifty-nine samples were positive for ReTV-variant2-RNA2-2F/2R, also with a detection rate of 100%. Eight PCR products were randomly selected for sequencing, revealing molecular variations of 99.0–100%. In summary, the ReTV-variant1 detection rate reached up to 35% (21/60), while the ReTV-variant2 detection rate was 100% (60/60). These results showed that the infection rate of ReTV-variant2 among *R. glutinosa* was higher than that of ReTV-variant1.

## 4. Discussion

In this study, we presented the complete sequences of ReTV through HTS, RT-PCR, and 5′ and 3′ RACE. HTS has become a popular method for rapidly detecting known and novel plant-infecting viruses [33,34]. Alfredo Diaz-Lara et al. [35] discovered grapevine enamovirus 2, a new member of the Genus *Enamovirus*, using HTS. HTS was utilized to obtain all contigs and compare them with other viruses in the NCBI database. In addition to the six viruses previously mentioned, we discovered 12 contigs that corresponded to viruses within the genus *Torradovirus*. Using RT-PCR and 5′ and 3′ RACE, the novel virus was identified. We followed the species classification criteria and compared the Pro-Pol regions and CPs of ReTV with those of other *torradoviruses*. The name “Rehmannia torradovirus virus” is proposed for this novel putative member of the genus *Torradovirus*.

The *Torradovirus* genus was established as containing two distinct groups based on TI and NTI members, determined by aa sequence identities of encoding putative proteins and the length of 3′ UTRs. The report indicates that torrado disease in Spain and Poland was generally observed in greenhouses or fields that were heavily infested with whiteflies. This led to suspicions that whiteflies might be insect vectors [36]. Three TI torradoviruses—ToTV, ToMarV, and ToChV—are transmitted by three whitefly species: T. *vaporariorum*, B. *tabaci*, and T. *abutilonea* (Haldeman) [37]. In the NTI group, CaTV is transmitted by aphids *Myzus persicae*, *M. persicae* biotype, and *Cavariella aegopodii* [38,39]. The transmission vectors of NTI *torradovirus* affecting cassava (CsTLV), lettuce (LNLCV), and motherwort (MYMoV) have not been identified. ReTV has been identified in *R. glutinosa* and is closely associated with SCLSV. As a new virus and host, future studies should aim to confirm its natural and experimental host ranges as well as vector transmission. 

Multiple viral infections within the same host plant are common in the field and often lead to synergism or antagonism among different viruses, usually with varied pathological outcomes [40]. Li et al. discovered that *tomato chlorosis virus* (ToCV) and *tomato yellow leaf curl virus* (TYLCV) mixed infections induced synergistic tomato disease, leading to a higher disease severity index and decreased stem heights and weights. Additionally, viral accumulation in ToCV and TYLCV mixed infected plants was higher than that in singly infected plants [41]. Infection with sweet potato feathery mottle virus and sweet potato chlorotic stunt virus leads to the development of sweet potato viral disease characterized by severe leaf symptoms and stunted plant growth [42]. ToTV is often (60% of the time) found together with *pepino mosaic virus* as well as in combination with other viruses, such as CMV, ToMV, *tomato spotted wilt virus*, and *tomato yellow leaf curl virus*. This observation led the authors to conclude that the symptoms of torrado may not solely be attributed to ToTV [43]. The ReTV isolates, which had been identified before this report, were mixed with ReMV, BBWV2, YoMV, TMGMV, CCYV, and CLVd. Further research into the molecular mechanisms of co-infection is necessary to explore new viruses. Disease prevention and control strategies provide a theoretical basis.

To compare all ten ReTV isolates, phylogenetic analysis and aa alignment of the Pro-Pol regions and CPs were conducted. This resulted in their clustering into two groups. We tentatively believe they were divided into two subspecies. These subspecies exhibited significant variations, all surpassing the species classification criteria. Ferriol I et al identified the cleavage site of the polyprotein of RNA2-ORF2 using the aa alignment [44]. The cleavage sites of a novel torradovirus, Burdock mosaic virus, were identified by aligning its aa sequence with those of other torradoviruses [19]. Therefore, it was identified the cleavage site of ReTVs-RNA2-ORF2 by aligning aa sequence with other viruses of the genus *Torradovirus*. The results showed distinct cleavage sites for the two subspecies in the MP and CPs. However, experimental validation is needed to determine the precise location of these cleavage sites between the putative CPs and MP. This demonstrates the significant molecular variations between the two subspecies, highlighting the need for focused future research on these variations. 

Furthermore, eight specific primers were developed to detect ReTV. The detection rates for ReTV1 ranged from 23.3–35%, with molecular variation between 90.6 and 100%. ReTV2 showed a detection rate of 100%, with molecular variation ranging from 98.0–100%. In summary, since the ReTV isolates represent the same virus, this new virus was detected in all 60 *R. glutinosa* samples. Therefore, it is speculated that ReTV is an important virus infecting *R. glutinosa* and that this virus warrants further study. 

This study, a novel Torradovirus-ReTV was found, though not without limitations. The area we sampled was mainly in Henan rather than all of China. The mode of viral transmission and the combination of infection need to be verified experimentally. Furthermore, the cleavage sites of this virus were identified by aligning aa sequences; accordingly, this needs to be verified experimentally.

## 5. Conclusions

In this study, we obtained the complete sequences of ReTV. The full-length RNA1 and RNA2 of ReTV-52 are 6939nt and 4569nt, respectively. However, the full-length RNA1 and RNA2 of ReTV-8 are 6889nt and 4662nt, respectively. The Pro-Pol regions of ReTV-52 and ReTV-8 exhibited aa homology ranges of 51.3–73.3% and 52.7–72.8%, respectively, compared with other viruses of the genus *Torradovirus*. Similarly, the CPs showed aa homology ranges of 37.1–68.1% and 36.8–67.5% for ReTV-52 and ReTV-8, respectively. Based on aa comparisons and phylogenetic analysis, the ten ReTV strains into two subspecies. Based on the detection of ReTV, it is speculated that it was the predominant virus infecting *R. glutinosa*.

## Figures and Tables

**Table 1:** Identity at the aa level (%) between the different *Torradoviruses* in the indicated regions with ReTV-52 (former) and ReTV-8 (latter).

Virus	Acronym	GenBank Accession (RNA1/RNA2)	Identity at the aa Level (%)					
			RNA1		RNA2			
			ORF1	Pro-Pol	ORF1	ORF2	MP	CPs
Motherwort yellow mottle virus	MYMoV	KM229700/ KM229701	34.9%/34.6%	56.0%/55.8%	18.8%/24.3%	32.6%/33.7%	22.7%/25.7%	38.2%/ 39.1%
Carrot torradovirus 1	CaTV1	KF533719/ KF533720	34.7%/34.4%	56.9%/54.9%	22.7%/24.9%	33.2%/33.9%	27.0%/27.7%	38.7%/ 39.1%
Codonopsis torradovirus A	CoTVA	MZ325520/ MZ325521	34.4%/34.3%	56.6%/56.2%	22.7%/23.8%	31.4%–31.5%	25.3%/24.3%	37.1%/ 37.1%
Squash chlorotic leaf spot virus	SCLSV	KU052530/ KU052531	54.6%/54.9%	73.3%/72.8%	47.0%/47.5%	62.2%/62.2%	52.3%/54.3%	68.1%/ 67.5%
Lettuce necrotic leaf curl virus	LNLCV	KC855266/ KC855267	34.7%/34.8%	55.1%/54.6%	18.2%/22.1%	33.2%/33.0%	26.3%/25.0%	38.7%/ 38.2%
Tomato torrado virus	ToTV	DQ388879/ DQ388880	31.7%/32.2%	51.3%/52.7%	22.7%/24.9%	33.9%/32.1%	27.0%/25.3%	38.1%/ 36.8%
Tomato marchitez virus	ToMarV	EF681764/ EF681765	33.3%/33.3%	56.2%/55.3%	24.3%/26.5%	34.5%/33.5%	29.7%/29.3%	38.1%/ 37.8%
Burdock mosaic virus	BdMV	OQ087134/ OQ087135	34.2%/34.6%	54.7%/56.3%	25.4%/26.0%	33.3%/33.2%	25.2%/25.5%	39.4%/ 40.0%
Fleabane yellow mosaic virus	FbYMV	OL979629/ OL979630	33.9%/34.2%	54.3%/54.1%	24.9%/24.9%	32.3%/32.4%	23.0%/23.3%	38.3%/ 38.4%
Tomato chocolate spot virus	ToCSV	GQ305131/ GQ305132	33.1%/33.0%	56.1%/55.2%	24.9%/25.4%	33.8%/33.4%	25.8%/26.7%	38.7%/ 38.0%

**Table 2:** Nucleotide sequence homology (%) in the corresponding regions of 10 ReTV genome RNA1 and RNA2 (%). Bold numbers indicate that the ReTV sequence has the highest or lowest consistency with other virus sequences.

Virus	ReTV-7	ReTV-8	ReTV-39	ReTV-40	ReTV-41	ReTV-44	ReTV-51	ReTV-52	ReTV-53	ReTV-57
ReTV-7		99.4%	99.3%	66.5%	67.0%	66.8%	99.2%	66.2%	66.3%	99.4%
ReTV-8	99.5%		99.4%	66.4%	67.0%	66.7%	99.2%	66.2%	66.3%	99.4%
ReTV-39	99.3%	99.2%		66.5%	67.0%	66.8%	99.2%	66.2%	66.4%	99.4%
ReTV-40	67.9%	67.9%	67.9%		91.8%	92.4%	66.4%	99.1%	99.2%	66.5%
ReTV-41	67.7%	67.6%	67.7%	93.9%		94.1%	66.9%	91.7%	91.8%	67.0%
ReTV-44	68.0%	67.9%	67.9%	94.3%	95.8%		66.6%	92.1%	92.2%	66.8%
ReTV-51	99.3%	99.2%	99.1%	67.9%	67.7%	67.9%		66.2%	66.3%	99.2%
ReTV-52	67.4%	67.3%	67.4%	93.9%	98.0%	94.5%	67.4%		99.5%	66.2%
ReTV-53	67.3%	67.3%	67.4%	93.8%	97.9%	94.3%	67.4%	99.7%		66.4%
ReTV-57	99.3%	99.3%	99.4%	67.9%	67.8%	67.9%	99.2%	67.5%	67.5%	

Percentages of amino acid sequences of RNA1 are shaded.

**Table 3:** Amino acid sequence homology (%) in corresponding regions of 10 ReTV genome Pro-Pol region and CPs. Bold numbers indicate that the ReTV sequence has the highest or lowest consistency with other virus sequences.

Virus	ReTV-7	ReTV-8	ReTV-39	ReTV-40	ReTV-41	ReTV-44	ReTV-51	ReTV-52	ReTV-53	ReTV-57
ReTV-7		99.6%	99.3%	84.1%	84.1%	83.7%	98.3%	83.2%	84.1%	99.6%
ReTV-8	99.3%		99.8%	84.5%	84.5%	84.1%	98.7%	83.7%	84.5%	100.0%
ReTV-39	99.2%	99.3%		84.3%	84.3%	83.9%	98.5%	83.4%	84.3%	99.8%
ReTV-40	83.3%	83.3%	83.3%		98.3%	98.9%	84.1%	98.7%	99.6%	84.5%
ReTV-41	82.6%	82.6%	82.7%	98.0%		98.9%	84.1%	96.9%	97.8%	84.5%
ReTV-44	83.3%	83.3%	83.3%	99.4%	98.0%		83.7%	97.6%	98.5%	84.1%
ReTV-51	99.4%	99.6%	99.7%	83.5%	82.8%	83.5%		83.2%	84.1%	98.7%
ReTV-52	83.7%	83.7%	83.7%	98.7%	98.7%	98.5%	84.0%		99.1%	83.7%
ReTV-53	83.4%	83.4%	83.4%	98.6%	98.6%	98.3%	83.7%	99.6%		84.5%
ReTV-57	99.3%	99.4%	99.6%	82.4%	82.7%	83.4%	99.9%	83.8%	83.5%	

Percentages of amino acid sequences in the Pro-Pol regions are shaded.

## Data Availability

Virus clones are available upon request. The raw data generated in this study are available in SRA NCBI (PRJNA1095028).

## Supplementary Materials

The following supporting information can be downloaded at: https://www.mdpi.com/article/10.3390/microorganisms12081643/s1, Supplementary Table S1. Primers used for complete sequencing of two variants of ReTV strains. Supplementary Table S2. Related information on 39 viruses included in the phylogenetic analysis. Supplementary Table S3. Specifics primers designed to discrimination the two variants of ReTV strains.
