# Supplementary material for: Genomic Analysis of a Novel Torradovirus “Rehmannia Torradovirus Virus”: Two Distinct Variants Infecting Rehmannia glutinosa
Source: Microorganisms. 2024 Aug 11;12(8):1643. doi: 10.3390/microorganisms12081643 (PMC11356386; doi:10.3390/microorganisms12081643)
Supplement: Supplementary file 1 [file microorganisms-12-01643-s001.zip › Supplementary Table S1.pdf]

Table S1. Primers used for complete sequencing of two variants of ReTV strains

Primer Name	Sequence(5' →3')	Position (nt)	Size (nt)	Used for
5'RACE	CTAATACGACTCACTATAGGGC	1-552	552	ReTV-variant1-RNA1
ReTV-variant1-RNA1-552R	CCACAGAGCTCGCCTCATACAGATC			
ReTV-variant1-RNA1-194F	TCTCAGATCAACTATTTCTGGTGC	194-828	634	
ReTV-variant1-RNA1-828R	GCTGCCATGGTCCCAACGGTAGAAG			
ReTV-variant1-RNA1-774F	CAGGACGGGCACGCCAAGCCATC	774-2883	2109	
ReTV-variant1-RNA1-2883R	GCAAGGTTCTTGGGCATGCTCTG			
ReTV-variant1-RNA1-2418F	GAGGATCTTAGAGGTGAAATGGAAT	2418-3627	1209	
ReTV-variant1-RNA1-3627R	GGTACATTCCCTAGATAAAACACCTG			
ReTV-variant1-RNA1-3529F	CGCCCCTTCAGGCGGCGCAGATAG	3529-4990	1461	
ReTV-variant1-RNA1-4990R	ACCCAGTACAAACATTCCTTAAC			
ReTV-variant1-RNA1-4441F	CCAATGGTTCTGCGATCTTGTGAGTG	4441-5190	749	
ReTV-variant1-RNA1-5190R	CAGTAACTTTCTCAATCCATATCA			
ReTV-variant1-RNA1-4959F	TGGGTGTCTGTTAAGGAATGCTTGT	4959-6003	1044	
ReTV-variant1-RNA1-6003R	GAATCCATGCTGCATGCAACGGAGTC			
ReTV-variant1-RNA1-5931F	GCAGCAAGCGAAGCTGGTGGCAATG	5931-6755	824	
ReTV-variant1-RNA1-6755R	GGACATATCTAAAGCAAGATCAAGC			
ReTV-variant1-RNA1-6491F	GGTGCTTGTTGTTGAGGCGCCAGAG	6491-6939	448	
oligodT	-------------			
5'RACE	CTAATACGACTCACTATAGGGC	1-311	311	ReTV-variant1-RNA2
ReTV-variant1-RNA2-311R	CCGTCTAACAGGAGGTGCCCTACAG			
ReTV-variant1-RNA2-276F	TTCTCTTTCACCCACACACACTGG	276-2118	1842	
ReTV-variant1-RNA2-2118R	GCCACAATATGAGCGCTAAATTC			
ReTV-variant1-RNA2-1839F	TGGGTTTCTCCTCCCAAATGTTTG	1839-2963	1124	
ReTV-variant1-RNA2-2963R	GCAAAGGCTCACTCACGGCAACA			
ReTV-variant1-RNA2-2858F	AGCTGGAATGGGAAGAATTTTCT	2858-3698	860	
ReTV-variant1-RNA2-3698R	ACAGCTCTGCATAGGTGTATGCCTC			
ReTV-variant1-RNA2-3194F	GGTGAGCTTCCTCTTTCTGCTG	3194-4091	897	
ReTV-variant1-RNA2-4091R	TTGTGAGGTGTTGTGAAGCGTCTG			
ReTV-variant1-RNA2-4032F	TGCAGTTCTTCTCCCTGGTAATG	4032-4569	537	
oligodT	--------------			
5'RACE	CTAATACGACTCACTATAGGGC	1-640	640	ReTV-variant2-RNA1
ReTV-variant2-RNA1-640R	CAATCGGACGTTCCAGCCAAG			
ReTV-variant2-RNA1-173F	AGACAGTTATTATCCTTCTTTG	173-1978	1805	
ReTV-variant2-RNA1-1978R	GTCCTCGTGTTCAGAAGAAACTG			
ReTV-variant2-RNA1-1778F	TGGCCTCTTGCTGACTTGTCTG	1778-3251	1473	
ReTV-variant2-RNA1-3251R	TGCCATAGGAAACACGAGTGAC			
ReTV-variant2-RNA1-3159F	GTGATTCAACAGAGGTGGGAGC	3159-4649	1490	
ReTV-variant2-RNA1-4649R	CATTCACGGCCCGCGCCCGTAT			
ReTV-variant2-RNA1-4431F	TAGTGAGTATTACAGAGACGC	4431-5681	1250	
ReTV-variant2-RNA1-5681R	AGCCTGGGTAAGAGGGAGAAAC			
ReTV-variant2-RNA1-5434F	GAAGCAAGGAACTCCTTGTGAG	5434-6654	1220	
ReTV-variant2-RNA1-6654R	CAAGCGGGATGTTGACAGACTC			

ReTV-variant2-RNA1-6511F<br>oligodT	TGATCTTGCCATAGATATGTCCACT<br>------------------	6511-6886	375	
5'RACE<br>ReTV-variant2-RNA2-225R	CTAATACGACTCACTATAGGGC<br>TACAAAGATACAGTAGGTGTTGAAC	1-225	225	ReTV-variant2-RNA2
ReTV-variant2-RNA2-30F<br>ReTV-variant2-RNA2-1663R	CATATATCTTCCCCAATCGTAGC<br>ATGGTTGGTTGAGCCATCATGTC	30-1663	1633	
ReTV-variant2-RNA2-1545F<br>ReTV-variant2-RNA2-3215R	CGTTGGCGGCCCTTAGAGAGCG<br>TGCCTCTTGCTCCATGCCATTA	1545-3215	1670	
ReTV-variant2-RNA2-3121F<br>ReTV-variant2-RNA2-3776R	CAGAGAGGCAAACTAATCCAGC<br>AGTGGCTACCGGTGGATGAGACAC	3121-3776	655	
ReTV-variant2-RNA2-3670F<br>ReTV-variant2-RNA2-4639R	GACTTGGGTACGGCAGTTTCTCG<br>TTTCGTTATCAAGTAGTAACTA	3670-4639	969	
ReTV-variant2-RNA2-4451F<br>oligodT	CCACTTGTGCGGCAGTCTGTGTAT<br>------------------	4451-4662	211	

Note: ReTV variant1 including ReTV-40, ReTV-41, ReTV-44, ReTV-52, and ReTV-53, and ReTV variant2 including ReTV-7, ReTV-8, ReTV-39, ReTV-51, and ReTV-57.
