# Supplementary material for: Genomic Analysis of a Novel Torradovirus “Rehmannia Torradovirus Virus”: Two Distinct Variants Infecting Rehmannia glutinosa
Source: Microorganisms. 2024 Aug 11;12(8):1643. doi: 10.3390/microorganisms12081643 (PMC11356386; doi:10.3390/microorganisms12081643)
Supplement: Supplementary file 1 [file microorganisms-12-01643-s001.zip › Supplementary Table S2.pdf]

Table S2 Related information on 39 viruses included in the phylogenetic analysis

Genera	Virus (isolate name)	Acronym	Accession code(RNA1/RNA2)
Torradovirus	Rehmannia torradovirus virus-7(R. glutinosa)	ReTV-7	OR453964/OR453969
Torradovirus	Rehmannia torradovirus virus-8(R. glutinosa)	ReTV-8	OR453963/OR453968
Torradovirus	Rehmannia torradovirus virus-39(R. glutinosa)	ReTV-39	OR453965/OR453970
Torradovirus	Rehmannia torradovirus virus-40(R. glutinosa)	ReTV-40	OR453959/OR453974
Torradovirus	Rehmannia torradovirus virus-41(R. glutinosa)	ReTV-41	OR453960/OR453975
Torradovirus	Rehmannia torradovirus virus-44(R. glutinosa)	ReTV-44	OR453958/OR453976
Torradovirus	Rehmannia torradovirus virus-51(R. glutinosa)	ReTV-51	OR453966/OR453971
Torradovirus	Rehmannia torradovirus virus-52(R. glutinosa)	ReTV-52	OR453962/OR453973
Torradovirus	Rehmannia torradovirus virus-53(R. glutinosa)	ReTV-53	OR453961/OR453977
Torradovirus	Rehmannia torradovirus virus-57(R. glutinosa)	ReTV-57	OR453967/OR453972
Torradovirus	motherwort yellow mottle virus(AD01)	MYMoV-AD01	KM29700/KM229701
Torradovirus	motherwort yellow mottle virus	MYMoV	NC035218/NC035220
Torradovirus	Codonopsis torradovirus A(SK)	CoTVA-SK	MZ325520/MZ325521
Torradovirus	Codonopsis torradovirus A	CoTVA	NC079055/NC079056
Torradovirus	lettuce necrotic leaf curl virus(5317015)	LNLCV-5317015	KC855266/KC855267
Torradovirus	lettuce necrotic leaf curl virus(JG3)	LNLCV-JG3	MW172270/MW172271
Torradovirus	carrot torradovirus 1	CaTV1	LC436363/LC436364
Torradovirus	carrot torradovirus 1(CTV-1)	CaTV1-CTV-1	KF533719/KF533720
Torradovirus	carrot torradovirus 1	CaTV1	NC025479/NC025480
Torradovirus	tomato marchitez virus(PRI-0601)	ToMarV-PRI-0601	EF681764/EF681765
Torradovirus	tomato marchitez virus(M)	ToMarV-M	KT756874/KT756875
Torradovirus	tomato chocolate spot virus	ToChSV	GQ305131/GQ305131
Torradovirus	tomato torrado virus(PRI-0301)	ToTV-PRI-0301	DQ388879/DQ388880
Torradovirus	tomato torrado virus(T795)	ToTV-T795	KX132808/KX132809
Torradovirus	tomato torrado virus	ToTV	NC009013/NC009032
Torradovirus	cassava torrado-like virus	CsTLV	OK040225/OK040226
Torradovirus	cassava torrado-like virus	CsTLV	NC079070/NC079069
Torradovirus	squash chlorotic leaf spot virus(Su12-10)	SCLSV-Su12-10	KU052530/KU052531
Torradovirus	squash chlorotic leaf spot virus	SCLSV	NC035221/NC035215
Sadwavirus	satsuma dwarf virus	SDV	AB009958/AB009959
Sadwavirus	satsuma dwarf virus(EHI)	SDV-EHI	MT344107/MT344108
Stralarivirus	strawberry latent ringspot virus	SLRSV	AY860978/AY860979
Stralarivirus	strawberry latent ringspot virus	SLRSV	NC006964/NC006965
Stralarivirus	lychnis mottle virus(Andong)	LycMoV	KR011032/KR011033
Cheravirus	cherry rasp leaf virus	CRLV	AJ621357/AJ621358
Cheravirus	apple latent spherical virus	ALSV	AB030940/AB030941
Cheravirus	apple latent spherical virus	ALSV	NC003787/NC003788
Cheravirus	currant latent virus(Hol9/6)	CuLV- Hol9/6	KT692952/KT692953
Cheravirus	currant latent virus	CuLV	NC029038/NC029036
