# Supplementary material for: Genomic Analysis of a Novel Torradovirus “Rehmannia Torradovirus Virus”: Two Distinct Variants Infecting Rehmannia glutinosa
Source: Microorganisms. 2024 Aug 11;12(8):1643. doi: 10.3390/microorganisms12081643 (PMC11356386; doi:10.3390/microorganisms12081643)
Supplement: Supplementary file 1 [file microorganisms-12-01643-s001.zip › Supplementary Table S3.pdf]

Table S3 Specifics primers designed to discrimination the two variants of ReTV strains

Primer name	Sequence (5' →3')	Position (nt)	Used for
ReTV-variant1-RNA1-1147F	GCTTTGCTGAGGCTACATACATAA	1147-1680	ReTV-variant1-RNA1
ReTV-variant1-RNA1-1680R	CCCTGTGGGACTATTAGAAGTG		
ReTV-variant1-RNA1-4364F	CCAGTTCCCTGAAGTGAATGAC	4364-5064	
ReTV-variant1-RNA1-5064R	TAGAAAGCCTCACGCATAGCC		
ReTV-variant1-RNA2-2936F	ATCTTTGTTGCCGTGAGTGAGCC	2936-3498	ReTV-variant1-RNA2
ReTV-variant1-RNA2-3498R	TCACCTTTGTAGAAGCCTGGAGC		
ReTV-variant1-RNA2-3081F	GGTCAAGCGGGTTAGACTTCTTC	3081-3747	
ReTV-variant1-RNA2-3747R	TTGAATCCAGGACCTGCACTTAT		
ReTV-variant2-RNA1-5756F	GATGTTACTACTGAGTCTATCGCCT	5756-6463	ReTV-variant2-RNA1
ReTV-variant2-RNA1-6463R	GCATCGTCCGACACTACTACTT		
ReTV2-variant2-RNA1-3629F	ATTCATCAAACTCTTACTGAGAC	3629-4139	
ReTV-variant2-RNA1-4139R	TCCTTTGGCAACCTTTCATCT		
ReTV-variant2--RNA2-3020F	GACTTTCACGCTCAAGAGCATTG	3020-3587	ReTV-variant2-RNA2
ReTV-variant2-RNA2-3587R	TTCCGAATTGGGACCAAGATTAA		
ReTV-variant2-RNA2-3092F	TGTGGCAAAGGGATCTAGTGGGT	3092-3739	
ReTV-variant2-RNA2-3739R	TAGGTGTAAGATTCTGCGGGTGG		
